# The case for investing in family planning in the Pacific: costs and benefits of reducing unmet need for contraception in Vanuatu and the Solomon Islands

**DOI:** 10.1186/1742-4755-10-30

**Published:** 2013-06-10

**Authors:** Elissa C Kennedy, Sean Mackesy-Buckley, Sumi Subramaniam, Andreas Demmke, Rufina Latu, Annette Sachs Robertson, Kabwea Tiban, Apisai Tokon, Stanley Luchters

**Affiliations:** 1 Centre for International Health, Burnet Institute, 85 Commercial Road, Melbourne, Victoria, Australia; 2 School of Public Health and Preventive Medicine, Monash University, Victoria, Australia; 3 Family Planning International, Wellington, New Zealand; 4 Independent consultant, population and development, Avarua, Cook Islands; 5 Health Systems Development, World Health Organisation, Port Vila, Vanuatu; 6 United Nations Population Fund Pacific Sub-Regional Office, Suva, Fiji; 7 International Planned Parenthood Federation East and Southeast Asia and Oceania Region, Suva, Fiji; 8 Ministry of Health, Port Vila, Vanuatu; 9 School of Public Health, University of the Witwatersrand, Johannesburg, South Africa; 10 International Centre for Reproductive Health, Department of Obstetrics and Gynaecology, Ghent University, Ghent, Belgium

**Keywords:** Family planning, Contraception, Women’s Health, Infant Health, Adolescent Fertility, Population, Economic Development

## Abstract

**Background:**

Unmet need for family planning in the Pacific is among the highest in the world. Better understanding of required investments and associated benefits of increased access to family planning in the Pacific may assist prioritisation and funding.

**Methods:**

We modelled the costs and associated health, demographic and economic impacts of reducing unmet need for family planning between 2010–2025 in Vanuatu and the Solomon Islands. Baseline data were obtained from census reports, Demographic and Health Surveys, and UN agency reports. Using a demographic modelling program we compared a scenario of “no change in unmet need” with two distinct scenarios: 1) all family planning needs met by 2020; and, 2) all needs met by 2050.

**Results:**

Meeting family planning needs by 2020 would increase prevalence of modern contraception in 2025 from 36.8 to 65.5% in Vanuatu and 28.5 to 37.6% in the Solomon Islands. Between 2010–2025 the average annual number of unintended pregnancies would decline by 68% in Vanuatu and 50% in the Solomon Islands, and high-risk births would fall by more than 20%, averting 2,573 maternal and infant deaths. Total fertility rates would fall from 4.1 to 2.2 in Vanuatu and 3.5 in the Solomon Islands, contributing to slowed population growth and lower dependency ratios. The direct cost of reducing unmet need by 2020 was estimated to be $5.19 million for Vanuatu and $3.36 million for the Solomon Islands between 2010–2025. Preventing unintended pregnancies would save $112 million in health and education expenditure.

**Conclusions:**

In small island developing states such as Vanuatu and the Solomon Islands, increasing investment in family planning would contribute to improved maternal and infant outcomes and substantial public sector savings.

## Background

The ability to decide freely the number, spacing and timing of children is a fundamental human right with proven benefits for the health of women and children. Reducing global unmet need for contraception would prevent around 30% of maternal deaths, reduce child mortality by up to 20%, and avert 36 million years of healthy life lost each year [1,2]. Additionally, family planning contributes to universal education, women’s empowerment, prevention of HIV, poverty reduction, and environmental sustainability, making it one of the most cost-effective global health and development interventions [3-6].

Despite these imperatives, progress towards universal access to family planning in the Pacific has been slow and inequitable. Many countries in this region report prevalence rates of modern methods of contraception well below the global average of 56% for less developed countries, and unmet need is among the highest in the world: between 8% and 46% of married women want to avoid pregnancy but are not using any method of family planning [6]. In some settings, more than half of all births are unintended, and as many as one in four adolescent girls have commenced childbearing [7].

Vanuatu and the Solomon Islands are made up of more than 1000 islands and are among the poorest in the Pacific. Less than 40% of women aged 15–49 use any method of family planning and between 11 and 30% have an unmet need for contraception [8-10]. Fertility rates remain above 4 and, with annual population growth rates over 2.3%, they will account for much of the expected population growth in the region [11].

To date there has been little published analysis of the potential costs and benefits of increasing access to family planning in small island developing states, including those of the Pacific. This lack of evidence has contributed to inadequate prioritisation and funding for family planning and slow progress towards universal access to reproductive health in this region [12]. With family planning recently re-positioned on the international development agenda [13], there is a need to assess the required investment and impact of meeting commitments to family planning in the Pacific.

In this study, we modelled the health, demographic and economic consequences of reducing unmet need in Vanuatu and the Solomon Islands and estimated the resources required to achieve this.

## Methods

We used the demographic modelling software *Spectrum 4*.*391* (Futures Institute, Glastonbury, CT, USA) to examine health, demographic and economic consequences of reducing unmet need for family planning in Vanuatu and the Solomon Islands. A detailed description of the program methodology and assumptions has been published elsewhere [14-17]. In brief, the program is based on a standard demographic cohort-component model and uses the proximate determinants of fertility framework to relate contraceptive use to total fertility rate (TFR) [18].

Baseline population projections (assuming no change in unmet need for family planning) were generated for Vanuatu and the Solomon Islands for the period 2010–2054. This projection period was chosen as both countries are predicted to reach replacement fertility by 2054 [19,20]. Two additional projections were generated separately for each country based on two hypothetical family planning scenarios: all needs met by 2020 (scenario one) and all needs met by 2050 (scenario two). Neither country is likely to achieve universal access to family planning by 2015 [12], so a target of 2020 was considered to be a best-case scenario. The additional target of 2050 was included to examine the impact of slower progress.

A panel of Pacific and international family planning and population experts and representatives from Ministries of Health (MOH) in both countries provided guidance concerning data and key assumptions.

### Data sources

The projections required base-year data for over 40 indicators of demography, health, determinants of fertility, family planning usage and costs, economy, and education. Definitions and estimates for key indicators are detailed in Table 1. The most recent estimates available were obtained from a range of sources including census reports, Demographic and Health Surveys (DHS), UN agency reports, and through consultation with MOH and key informants in each country.

**Table 1:** **Key population**, **reproductive health**, **and economic estimates for the base year** (**2010**) **for Vanuatu and the Solomon Islands**

**Indicator**	**Vanuatu**	**Solomon Islands**
Population size (number) [19,20]	239,000	515,970
Annual population growth rate (%) [19,20]	2.3	2.6
Total fertility rate [19,20]	4.1	4.1
Adolescent fertility rate (births per 1000 women aged 15–19) [19,20]	66	62
Proportion of women aged 15–49 married or in union (%) [19,20]	65	61
Median months postpartum insusceptibility (months) [9]	11	11
Proportion of unwanted pregnancies ending in induced abortion (%) [21]	33	33
Proportion of women aged 45–49 who have never given birth (%) [19,20]	7	7
Contraceptive prevalence rate, all methods (%) [9,10]	38	35
Contraceptive prevalence rate, modern methods (%) [9,10]	37	27
Proportion of women aged 15–49 and married or in union with unmet need for family planning (%) [8,9]	30	11
Proportion of births with any avoidable risk† (%) [9]	55	55
Maternal mortality ratio (deaths per 100,000 live births) [19,22]	110	162
Infant mortality rate (deaths per 1000 live births) [19,20]	21	23
Gross domestic product per capita (US$) [23]	2,526	1,147
Annual expenditure per primary student (US$) [24,25]	363	192
Annual expenditure per secondary student (US$) [10,24]	1,146	462
Annual health expenditure per capita (US$) [26]	104	72

† Births to women aged <18 or >34 years; births spaced <24 months apart; birth order 4 or more.

For this analysis, unmet need for family planning was defined as the percentage of fecund women of reproductive age (15–49 years) who are married or in consensual union, who want no more children or want to delay pregnancy by two years or more, and are not using any method of family planning (including traditional methods). This includes pregnant or amenorrhoeic women whose last pregnancy was mistimed or unwanted [27]. The estimate of unmet need for the Solomon Islands (11% (95% confidence interval (95% CI): 10-12%)) was sourced from the 2006–2007 DHS [9]. Data for unmet need in Vanuatu are limited. Following recommendation from the expert panel, a 1995 UNFPA estimate of unmet need for birth limiting was included (30% (95% CI: 28-33%)) [8].

Base year estimates of contraceptive method mix (including oral contraceptive pill, injectable, implant, intrauterine device, male and female sterilisation, male condom, female condom and other vaginal methods, and traditional methods) were obtained from DHS data and the 2007 UNICEF Vanuatu Multiple Cluster Indicator Survey [10]. Contraceptive effectiveness was based on estimates of first-year unintended pregnancy rates for each method as commonly used, provided by the World Health Organisation (WHO) [28].

We assumed a service-delivery perspective for this analysis. The direct costs (government and non-government) of providing family planning per contraceptive method (per couple-year of protection for short-acting methods and per acceptor for long acting methods) were calculated from cost estimates of: commodities, supplies and equipment procurement; shipping, storage and distribution; and staff costs for counselling, method provision and follow-up. Commodity, equipment, transport and storage costs were obtained directly from the Pacific Sub-Regional Office of UNFPA (the major supplier of family planning commodities in Vanuatu and the Solomon Islands), International Planned Parenthood Federation (IPPF) East and Southeast Asia and Oceania Region (the major non-government provider) and MOH of each country. Staff costs were based on estimates of average staff salaries and time spent per client per method obtained from MOH and IPPF clinics. Other non-government and private providers as well as out-of-pocket family planning expenditure were not included due to lack of reliable data. All costs were converted to US dollars based on the official nominal exchange rate and adjusted to a 2010 price year [23].

### Assumptions

Thirty of the 40 model inputs required yearly estimates for the entire projection period. All base year inputs and assumptions (except for the proportion of women with unmet need) were the same for the baseline and two hypothetical projections for each country.

Unmet need remained constant for the baseline projection. In the other two projections the reduction in unmet need was ‘front loaded’ commencing in 2010, assuming a more rapid initial increase in contraceptive prevalence [29], with all needs met by 2020 (scenario one) and by 2050 (scenario two). Due to the lack of age-disaggregated data, the reduction in unmet need was assumed to be evenly distributed across all age groups.

Estimates for proximate determinants of fertility remained constant. Projected contraceptive method mix for both countries was adjusted to take into account the planned introduction of contraceptive implants and to adjust for the current high reliance on oral contraceptives in Vanuatu and female sterilisation in the Solomon Islands. The adjusted method mix was estimated from global trend data, the average method mix for the Pacific region, and following consultation with regional and international family planning experts [29-31]. In brief, the prevalence of long-acting and permanent methods of contraception was increased in Vanuatu, while the current low prevalence of traditional methods remained constant. In the Solomon Islands the prevalence of traditional methods was projected to halve by 2054, while other methods were adjusted to the Pacific average. In both countries the prevalence of intrauterine devices and condoms remained constant. Source mix and direct costs per method also remained constant.

Age-specific fertility rates were projected to reach the average of Australia, New Zealand, France and USA by 2054 as per the methodology used by the Statistics and Demography Programme of the Secretariat of the Pacific Community. Future life expectancy was calculated using the UN models for mortality improvement assuming medium gains [32]. Economic growth, health and education expenditure were assumed to reach the average for East Asia and the Pacific by 2054 based on the most recent data from the World Bank [23], UNESCO [33], WHO [26] and the International Monetary Fund [34,35].

### Outcome measures and data analysis

Primary outcomes included contraceptive prevalence and number of users per method, family planning costs, health outcomes (unintended pregnancies, induced abortions, total births, births with any avoidable risk, and maternal and infant deaths), TFR, population growth, dependency ratio, and annual public sector health and education expenditure. The program was used to project these outcomes, with analysis restricted to the time period 2010–2025. The program methodology has been described elsewhere [15-17], however, in brief, unintended pregnancies are calculated from pregnancies due to contraceptive failure and those to women of reproductive age (married or in union) with unmet need for contraception. Maternal deaths per year are calculated from the number of deaths associated with both wanted and unwanted pregnancies:

$$\begin{array}{ll} \mathrm{Maternal}\;\mathrm{deaths}= & \left(\mathrm{BW}\;X\;\mathrm{MMR}/100,000\right)\\ & +\left(\mathrm{BNW}\;X\;\mathrm{MMR}/100,000\right) \end{array}$$

where *BW* is the number of wanted births and *BNW* the number of unwanted births. Infant deaths are calculated using an adjusted infant mortality rate (IMR) related to risky births:

$$\begin{array}{ll} \mathrm{IMR}\left(t\right)=\mathrm{IMR}\left(0\right)- & \left(\%\mathrm{risky}\;\mathrm{births}\left(0\right)-\%\mathrm{risky}\;\mathrm{births}\left(t\right)\right)\\ & /\%\mathrm{risky}\;\mathrm{births}\left(0\right)\;X\;\mathrm{IMR}\left(0\right) \end{array}$$

where *t* is the target year and *0* base year. Avoidable high-risk births are defined as those that occur at extremes of maternal age (younger than 18 and more than 34 years), are spaced less than 24 months apart, or are high parity (birth order 4 and higher) [27]. Infant deaths are then calculated by multiplying the total number of births by the adjusted IMR.

Projected outcome data for each model for the period 2010–2025 were extracted and analysed using Microsoft Excel (Microsoft Corp, Redmond, WA, USA). Future costs and health effects were discounted by 3% per year [36]. The impact of reducing unmet need by 2020 and 2050 was compared to the baseline model for each outcome of interest. All costs are reported in US dollars.

## Results

### Contraceptive prevalence

Meeting all needs for family planning by 2020 would increase the contraceptive prevalence rate (all methods) from 38.4% to 68.4% in Vanuatu, resulting in 38,164 users by 2025, 16,739 more than if unmet need remained constant (Table 2). Ninety-six per cent of users would rely on modern methods, with a third using a long-acting or permanent method. Contraceptive prevalence in the Solomon Islands would increase from 34.6% to 45.6%. Of the additional 10,922 users, 82% would be using a modern method and 47% a long-acting or permanent method.

**Table 2:** **Projected outcomes by 2025 for contraceptive use and fertility for the baseline model** (**constant unmet need**) **and two scenarios** (**all needs met by 2020 and all needs met by 2050**) **in Vanuatu and Solomon Islands**

**Contraceptive use and fertility**	**Vanuatu**			**Solomon Islands**		
	**Constant unmet need**	**All needs met by 2050**	**All needs met by 2020**	**Constant unmet need**	**All needs met by 2050**	**All needs met by 2020**
Total contraceptive users (all methods)	21,425	34,599	38,164	34,353	42,949	45,275
Contraceptive prevalence rate (all methods) %	38.4	62.0	68.4	34.6	43.3	45.6
Contraceptive prevalence rate (modern methods) %	36.8	59.4	65.5	28.5	35.7	37.6
Contraceptive prevalence rate (long-acting or permanent) %	12.8	20.6	22.8	16.3	20.4	21.5
Total fertility rate	4.0	2.6	2.2	4.1	3.7	3.5
Adolescent fertility rate (births per 1000 females aged 15–19)	59.1	37.8	32.2	56.8	50.6	48.9

### Reproductive health

By 2025, the rate of unintended pregnancies in Vanuatu would fall from 76 per 1000 women aged 15–49 if unmet need remained constant to 12 per 1000 if all needs were met by 2020, averting an average of 3,120 unintended pregnancies and 2,090 unplanned births every year (Table 3). In the Solomon Islands, 2,075 unintended pregnancies and 1,388 unplanned births would be prevented each year, reducing the unintended pregnancy rate from 31 per 1000 to 12 per 1000 women aged 15–49. Our projections indicate that meeting all family planning needs by 2020 would reduce the number of abortions in Vanuatu by 68% and by half in the Solomon Islands. By preventing unintended pregnancies, there would be an estimated three fewer maternal deaths per year in each country between 2010 and 2025.

**Table 3:** **Projected health outcomes for women and infants**, **average per year between 2010 and 2025**, **for the baseline model** (**constant unmet need**) **and two scenarios** (**all needs met by 2020 and all needs met by 2050**) **in Vanuatu and Solomon Islands**

**Projected average per year (2010–2025)**	***Vanuatu***					***Solomon Islands***				
	**Constant unmet need**	**All needs met by 2050**		**All needs met by 2020**		**Constant unmet need**	**All needs met by 2050**		**All needs met by 2020**	
		**Number**	***% reduction in outcome***	**Number**	***% reduction in outcome***		**Number**	***% reduction in outcome***	**Number**	***% reduction in outcome***
Total pregnancies	10,102	7,137	*29*	5,751	*43*	20,286	18,281	*10*	17,340	*15*
Unintended pregnancies	4,517	2,417	*47*	1,397	*69*	4,147	2,742	*34*	2,072	*50*
Induced abortions	1,491	798	*47*	461	*68*	1,368	905	*34*	684	*50*
Total births	7,298	5,411	*26*	4,542	*38*	16,280	15,000	*8*	14,402	*12*
Unintended births and miscarriages	3,026	1,620	*47*	936	*69*	2,778	1,840	*34*	1,390	*50*
Birth with any avoidable risk^‡^	4,049	2,530	*38*	1,940	*52*	8,922	7,720	*13*	7,185	*20*
Maternal deaths	7	5	*26*	4	*39*	26	24	*8*	23	*12*
Infant deaths	192	117	*39*	89	*54*	375	324	*13*	302	*20*

‡ Births to women aged <18 or >35 years; births spaced < 18 months apart; birth order 4 or more.

The annual number of high-risk births would decrease by 54% and 20% in Vanuatu and the Solomon Islands respectively. Meeting all needs in Vanuatu would reduce the number of adolescent births in 2025 by 46%, lowering the adolescent fertility rate in 2025 from 59 births per 1000 girls aged 15–19 in the base model to 32 births per 1000 girls (Table 2). Meeting all needs in the Solomon Islands would reduce the adolescent fertility rate from 57 to 49 births per 1000 girls, contributing to improved maternal and perinatal outcomes. In Vanuatu, the IMR would fall from 21 to 15 deaths per 1000 live births, allowing the country to meet its Millennium Development Goal 4 target by 2016 [37]. In the Solomon Islands, the IMR would decline from 23 to 20 deaths per 1000 live births, averting an average of 73 deaths per year between 2010 and 2025.

### Population and demographic impacts

Assuming no change in unmet need, our projections suggest that TFR would remain around 4.1 in both countries over the next 16 years compared with 2.2 in Vanuatu and 3.5 in the Solomon Islands if all needs are met by 2020. Annual population growth would slow from 2.5% to 1.4% in Vanuatu and to 2.2% in the Solomon Islands by 2025. In both countries, satisfying the demand for family planning would lower the youth dependency ratio from 67 dependents (0–14 years) per 100 people of working age (15–59 years) to 39 and 58 in Vanuatu and the Solomon Islands respectively.

### Costs

**Figure 1:**
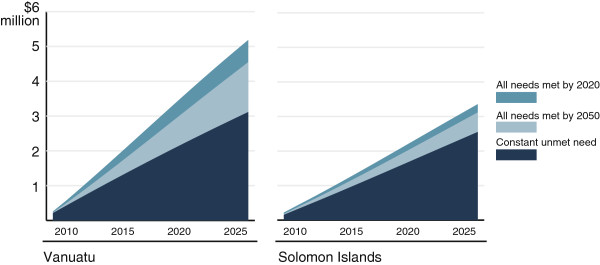
**Cumulative family planning costs (modern methods) between 2010–2025 for the baseline model (constant unmet need) and two scenarios (all needs met by 2020 and all needs met by 2050) in Vanuatu and Solomon Islands (US$ millions).**

The direct service delivery costs of reducing unmet need by 2020 in Vanuatu would be $324,282 per year on average between 2010 and 2025, a total of $5.19 million over the next 16 years (Figure 1). Meeting all family planning needs by 2020 would cost on average $13.56 per user, or $1.47 per capita per year. It would cost $104 to prevent an unintended pregnancy and $3,928 to avert the death of a woman or infant. In the Solomon Islands, an average of $209,885 per year would be required to meet the direct costs of reducing unmet need, a total of $3.36 million between 2010 and 2025. Addressing unmet need by 2020 would cost $7.46 per user or $0.40 per capita per year: $101 to prevent an unintended pregnancy and $2762 to avert a maternal or infant death. Even if unmet need and contraceptive prevalence remained constant, total family planning expenditure would continue to increase in both countries due to population growth resulting in an increase in the total number of women of reproductive age.

**Figure 2:**
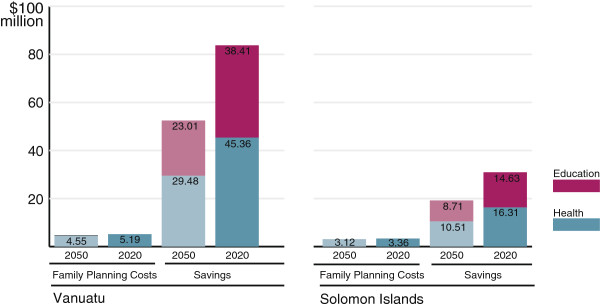
**Projected family planning costs and health and education savings (US$ millions) of meeting all family planning needs by 2050 and 2020 compared with no change in unmet need for the period 2010–2025.**

Preventing unwanted births would result in substantial public sector savings. Between 2010 and 2025, meeting all family planning needs in Vanuatu would save $45 million in health and $38 million in education expenditure, resulting in a net saving of $82 million. In the Solomon Islands, $30 million would be saved between 2010 and 2025: $16 million in health and $15 million in education costs (Figure 2).

### Sensitivity analysis

One-way sensitivity analyses were conducted to test the robustness of estimates of averted unintended pregnancies and deaths, family planning costs and public sector savings to changes in key assumptions (Table 4 and Table 5). Under alternative assumptions of unmet need (based on the 95% CI), constant contraceptive method mix (based on current mix), constant age-specific fertility rates, and family planning costs (+/−25%) the total number of averted events (from 2010–2025) varied between 46,602 and 54,912 unintended pregnancies and 1,244 to 1,433 deaths in Vanuatu with costs per averted event ranging from $78 to $130 (unintended pregnancy) and $2,946 to $4,910 (deaths). In the Solomon Islands, estimates for averted events ranged from 30,191 to 36,203 unintended pregnancies and 1,109 to 1,321 deaths, with a cost of between $78 and $126, and $2,071 to $3,452 per averted unintended pregnancy and death respectively.

**Table 4:** **Sensitivity analysis**: **Vanuatu**

**Key alternative assumptions**	***Vanuatu***											
	**All needs met by 2050**						**All needs met by 2020**					
	**Total number of unintended pregnancies averted 2010-2025**	**Cost per unintended pregnancy averted (US$)**	**Total number of maternal and infant deaths averted 2010-2025**	**Cost per death averted (US$)**	**Total family planning costs 2010-2025 (US$)**	**Total public sector savings 2010**–**2025 (US$)**	**Total number of unintended pregnancies averted 2010**-**2025**	**Cost per unintended pregnancy averted (US$)**	**Total number of maternal and infant deaths averted 2010–2025**	**Cost per death averted (US$)**	**Total family planning costs 2010–2025 (US$)**	**Total public sector savings 2010–2025 (US$)**
Base case	33,602	$135	950	$4,790	$4,550,755	$51,054,348	49,922	$104	1,321	$3,928	$5,188,506	$81,696,290
Unmet need for contraception (28 – 33%)	31,353 – 37,004	$127 – $142	891 – 1,037	$4,527 –$5,000	$4,455,206 – $4,694,071	$47,331,683 – $56,739,129	46,602 – 54,912	$98 - $108	1,244 – 1,433	$3,766 – $4,059	$5,049,667 – $5,396,741	$75,778,322 – $90,721,234
Constant rate of reduction of unmet need	11,009	$327	338	$10,663	$3,604,082	$12,840,011	39,097	$122	1051	$$4,428	$4,759,294	$49,176,915
Constant contraceptive method mix	33,252	$138	949	$4,824	$4,578,332	$50,916,264	49,400	$106	1,321	$3,955	$5,223,927	$81,481,832
Constant age-specific fertility rate	33,573	$136	949	$4,795	$4,550,770	$51,070,751	49,883	$104	1,320	$3,931	$5,188,521	$81,730,979
Direct family planning costs +/−25%	33,602	$102 – $169	950	$3,593 – $5,988	$3,413,066 – $5,688,444	$50,695,941 –$51,412,755	49,922	$78 -– $130	1,321	$2,946 – $4,910	$3,891,379 – $6,485,632	$81,178,445 – $82,214,135
Recurrent public sector expenditure +/−25%	33,602	$135	950	$4,790	$4,550,755	$37,932,354 –$64,176,342	49,922	$104	1,321	$3,928	$5,188,506	$60,754,373 –$102,638,206
Discounting (0 – 5%)	28,366 – 44,103	$131 – $138	804 – 1241	$4,644 – $4,901	$3,940,312 – $5,762,703	$40,726,168 – $72,681,695	42,527 – 64,658	$101 – $106	1,128 – 1,705	$3,843 –$3,989	$4,500,054 – $6,552,595	$65,372,266 – $115,790,286

**Table 5:** **Sensitivity analysis**: **Solomon Islands**

**Key alternative assumptions**	***Solomon Islands***											
	**All needs met by 2050**						**All needs met by 2020**					
	**Total number of unintended pregnancies averted 2010-2025**	**Cost per unintended pregnancy averted (US$)**	**Total number of maternal and infant deaths averted 2010-2025**	**Cost per death averted (US$)**	**Total family planning costs 2010**–**2025 (US$)**	**Total public sector savings 2010–2025 (US$)**	**Total number of unintended pregnancies averted 2010-2025**	**Cost per unintended pregnancy averted (US$)**	**Total number of maternal and infant deaths averted 2010-2025**	**Cost per death averted (US$)**	**Total family planning costs 2010–2025 (US$)**	**Total public sector savings 2010–2025 (US$)**
Base case	22,479	$139	841	$3,708	$3,118,381	$18,661,773	33,201	$101	1,216	$2,762	$3,358,160	$30,129,839
Unmet need for contraception (11 – 12%)	20,446 – 24,506	$129 – $150	767 – 915	$3,464 – $3,999	$3,066,994 – $3,169,767	$16,921,477 – $20,411,314	30,191 – 36,203	$95 – $109	1,109 – 1,321	$2,598 – $2,962	$3,284,998 – $3,431,316	$27,425,861 – $32,947,475
Constant rate of reduction of unmet need	7,393	$373	285	$9,681	$2,759,065	$4,602,740	26,106	$123	960	$3,355	$3,221,007	$17,825,146
Constant contraceptive method mix	21,952	$133	840	$3,489	$2,930,505	$18,601,170	32,456	$97	1,213	$2,603	$3,157,995	$30,034,687
Constant age-specific fertility rate	22,521	$138	843	$3,699	$3,118,376	$18,672,780	33,257	$101	1,218	$2,757	$3,358,155	$30,144,376
Direct family planning costs +/−25%	22,479	$104 – $173	841	$2,781 – $4,635	$2,338,786 – $3,897,976	$18,520,429 – $18,803,118	33,201	$78 – $126	1,216	$2,071 – $3,452	$2,518,620 – $4,197,701	$29,928,549 – $30,331,128
Recurrent public sector expenditure +/−25%	22,479	$139	841	$3,708	$3,118,381	$13,854,987 – $23,468,562	33,201	$101	1,216	$2,762	$3,358,160	$22,396,091 – $37,863,588
Discounting (0 – 5%)	18,948 – 29,572	$134 – $142	711 – 1,104	$3,579 – $3,797	$2,699,916 – $3,950,801	$14,915,362 – $26,491,813	28,247 – 43,088	$98 – $103	1,036 – 1,573	$2,696 – $2,812	$2,913,710 – $4,241,273	$24,161,801 – $42,568,107

The most favourable estimates were calculated where there was no discounting of health effects or costs in both countries. The largest unfavourable effect on projected estimates was associated with the alternative assumption regarding the rate of reduction in unmet need. A constant (linear) reduction in unmet need substantially reduced the number of averted events and increased costs per averted event, most notably for the 2050 scenario. This effect is largely explained by the lower contraceptive prevalence rate (CPR) achieved by 2025 under this assumption: 49.7% in Vanuatu and 38.7% in the Solomon Islands (for all needs met by 2050). All estimates under alternative assumptions demonstrated health and economic benefits associated with reducing unmet need (compared to the baseline projection) and meeting this need by 2020 would result in larger benefits than slower progress.

## Discussion

This study examined the benefits and associated costs of reducing unmet need for family planning in small island developing states with low contraceptive prevalence. Our analysis suggests that reducing unmet need for family planning in Vanuatu and the Solomon Islands would have significant health benefits for women and infants. By substantially reducing unintended pregnancies and high-risk births, including those to adolescent girls, more than 1,300 deaths in Vanuatu and 1,200 in the Solomon Islands would be averted over the next 16 years.

Additionally, reducing unwanted pregnancy would have considerable demographic and economic benefits. Youth dependency would decrease by 12% in the Solomon Islands and 42% in Vanuatu, with limited data in Vanuatu indicating that households with fewer dependents have higher rates of school enrolment and increased wealth [38]. Fertility decline and reduced dependency have been demonstrated to contribute to improved opportunities for women and both immediate and long term economic gains for households and countries [4,39]. With 60% of the population aged under 25 years there is an opportunity for both countries to take advantage of the potential demographic dividend associated with rapid fertility decline [40]. Combined with appropriate investment in education and employment, this transition has been credited with contributing to economic development in other regions [41]. Increased funding for family planning would also result in significant public sector savings. For every dollar spent to reduce unmet need by 2020 between $9-16 could be saved on health and education, making development goals more attainable and more affordable.

Increased investment in family planning would be required over the next 16 years to achieve these outcomes. Our analysis estimates the direct costs of reducing unmet need by 2020 to be $14 and $7 per user per year in Vanuatu and the Solomon Islands respectively, with between $101 and $104 required to avert an unintended pregnancy. These projections are higher than global estimates that suggest the average cost per user of reducing unmet need in developing regions would be $3.40 (although there is considerable regional variation), with $28-30 required to prevent an unintended pregnancy [2]. The base-year direct costs calculated for this analysis are much higher than previous estimates for Asia, Africa and Latin America [42]. The high cost of service delivery is well noted in the Pacific, where transport costs, weak infrastructure and small, geographically dispersed populations pose particular challenges [43]. Such settings have little potential to benefit from economies of scale [44]. However, with total health expenditure in 2009 estimated at $37.72 million in the Solomon Islands and $25.22 million in Vanuatu, investing between $210,000 and $324,000 per year to meet family planning needs would seem a comparatively small increment [26].

Meeting all family planning needs by 2020 would result in an average annual increase in CPR of 1 percentage point per year in the Solomon Islands and 3 percentage points in Vanuatu. Given the global average for annual increase in CPR is around 1 percentage point [29-31], this scenario for Vanuatu is ambitious, and the alternative scenario of meeting all needs by 2050 (less then 1 percentage point per year) may present a more realistic goal. Meeting all family planning needs by 2050 would cost $638,000 and $240,000 less than achieving this goal by 2020 in Vanuatu and the Solomon Islands respectively, but would also result in fewer lives saved, fewer unintended pregnancies averted, and less savings to health and education sectors.

This analysis has several limitations. The validity of our estimates depends on the underlying methodology of the software program in addition to the quality of input data and assumptions. The software program is rigorously reviewed and updated in collaboration with external experts such as the Child Health Epidemiology Reference Group and the UNAIDS Reference Group on Estimates, Models and Projections, and has been used previously to examine public health impacts of family planning [45].

Unmet need for family planning is difficult to accurately measure [46]. The Vanuatu estimate for unmet need is based on secondary analysis by UNFPA and was restricted to unmet need for birth limiting. No recent data for Vanuatu are available, although consultation with Vanuatu and Pacific family planning experts indicated that this figure is a realistic estimate. Were Vanuatu’s unmet need substantially lower than the estimate used for this analysis then the health, demographic and economic benefits would be smaller than our findings. The current estimate for the Solomon Islands is likely to significantly underestimate the true demand for family planning. The recent DHS reported that as many as 57% of all births are unintended, suggesting that a substantial proportion of women who want to avoid pregnancy are not using an effective method of contraception [9]. Therefore we are likely to have underestimated the true cost and impact of meeting all needs for family planning in the Solomon Islands.

Further, we did not account for a potential future increase in unmet need, likely with improved female education and increasing community awareness [47]. While we assumed a more rapid increase in CPR at the beginning of the projection, we did not adjust for an initial increase in unmet need which may occur during the early expansion of family planning services, particularly in the context of low CPR [48]. These factors could be expected to increase the investment required but may also increase the potential health and demographic benefits. Other influences on fertility, such as female education, were not included in our model. Additionally, the two family planning scenarios assumed that all women with unmet need would become family planning users (traditional or modern methods), however it is noted that even in settings with high CPR there are likely to still be women with an unmet need for family planning due to factors such as personal or partner objection, religious or socio-cultural opposition, inadequate knowledge, or lack of access to acceptable methods [48,49].

Our analysis does not estimate all the costs associated with reducing unmet need. Costs related to increasing community awareness and demand, improving quality of services, strengthening health systems, or reaching populations with poor access were not included. These factors are difficult to quantify but may make up more than half of the total costs of increasing access to family planning particularly in small island states where considerable geographical and socio-cultural challenges exist [50]. In addition, costs remained constant throughout the projection period, so do not reflect potential changes in commodity procurement and/or transport costs. These estimates are therefore likely to underestimate the true investment required.

The relatively small number of live births and maternal and infant deaths makes the projected reduction in maternal and infant mortality difficult to interpret. However, our estimates that between 12-39% of maternal deaths and 20-54% of infant deaths could be averted by increasing access to contraception are similar to recent studies examining the global impact of contraception on maternal and child health [1].

## Conclusions

Despite the comparatively high costs associated with commodity supply and service delivery, investing in family planning in Vanuatu and the Solomon Islands would have substantial health, population and economic benefits, contributing to fewer unintended pregnancies, reduced maternal and infant mortality and significant savings in health and education expenditure. The international community recently pledged to meet the needs of an additional 120 million women and girls by 2020 [51]. There are considerable health, development and human rights imperatives to ensure that those in the Pacific are not overlooked.

## Abbreviations

CPR: Contraceptive prevalence rate; DHS: Demographic and health survey; IMR: Infant mortality rate; IPPF: International planned parenthood federation; MM: Maternal mortality ratio; MOH: Ministry of health; TFR: Total fertility rate; UNESCO: United Nations Educational, Scientific and Cultural Organisation; UNFPA: United Nations Population Fund; WHO: World Health Organisation.

## Competing interests

The authors declare that they have no competing interests.

## Authors’ contributions

EK, SMB and SS designed the study. Data collection was done by SMB and EK with input from AD, RL, AR, KT and AT. Data were analysed by EK and SMB with input from AD and SL. Data were interpreted by EK, SMB, SS and SL with additional input from AD, RL, AR, KT and AT. The manuscript was written by EK and feedback provided by all authors. All authors approved the final version of the manuscript for publication.
